# Selective adsorption of Hg(ii) with diatomite-based mesoporous materials functionalized by pyrrole–thiophene copolymers: condition optimization, application and mechanism†

**DOI:** 10.1039/d2ra05938j

**Published:** 2022-11-18

**Authors:** Yu Zhou, Zheng Zeng, Yongfu Guo, Xinyu Zheng

**Affiliations:** Department of Municipal Engineering, Suzhou University of Science and Technology Suzhou 215009 China yongfuguo@163.com xinyuzheng@mail.usts.edu.cn; Jiangsu Collaborative Innovation Center of Technology and Material of Water Treatment Suzhou 215009 Jiangsu China

## Abstract

A novel diatomite-based mesoporous material of MCM-41/co-(PPy-Tp) was prepared with MCM-41 as carrier and functionalized with the copolymer of pyrrole and thiophene. The physicochemical characteristics of the as-prepared materials were characterized by various characterization means. The removal behaviour of Hg(ii) was adequately investigated *via* series of single factor experiments and some vital influence factors were optimized *via* response surface methodology method. The results exhibit that diatomite-based materials MCM-41/co-(PPy-Tp) has an optimal adsorption capability of 537.15 mg g^−1^ towards Hg(ii) at pH = 7.1. The removal process of Hg(ii) onto MCM-41/co-(PPy-Tp) is controlled by monolayer chemisorption based on the fitting results of pseudo-second-order kinetic and Langmuir models. In addition, the adsorption of Hg(ii) ions onto MCM-41/co-(PPy-Tp) is mainly completed through forming a stable complex with N or S atoms in MCM-41/co-(PPy-Tp) by electrostatic attraction and chelation. The as-developed MCM-41/co-(PPy-Tp) displays excellent recyclability and stabilization, has obviously selective adsorption for Hg(ii) in the treatment of actual electroplating wastewater. Diatomite-based mesoporous material functionalized by the copolymer of pyrrole and thiophene exhibits promising application prospect.

## Abstract 2

The diatomite-based MCM-41/co-(PPy-Tp) has a Langmuir adsorption capacity of 533.57 mg g^−1^ towards Hg(ii). Synthesized adsorbent has high selectivity for Hg(ii) in the treatment of electroplating wastewater.

## Graphical abstract

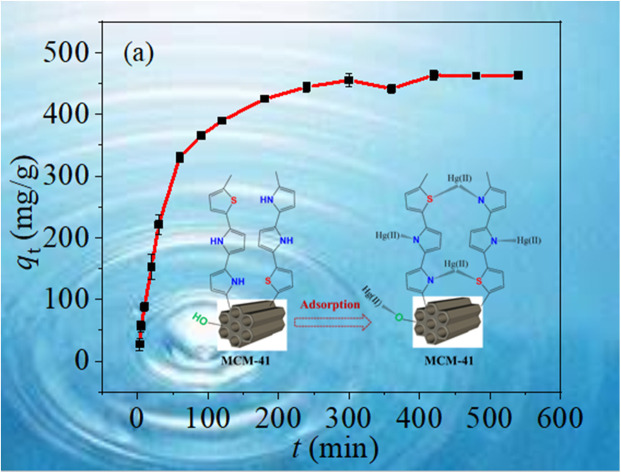

## 1. Introduction

The development of modern industrialization has led to the release of large amounts of heavy metals into the water bodies, causing serious damage to the environment and public health.^1^ Especially mercury, as one of the most toxic heavy metals, has non-biodegradable, persistent and bioaccumulative characteristics.^2^

A large number of studies have shown that inorganic mercury in water (mainly divalent mercury Hg(ii)) can easily be converted into more deadly methylmercury under the action of certain bacteria. Methylmercury is a highly effective neurotoxin that can cause a series of diseases, such as kidney failure, brain damage and endocrine disorders.^3^ Therefore, how to remove mercury ions from water environment has become a major problem.^4^

In recent decades, methods such as chemical precipitation, membrane separation, ion exchange and adsorption have been commonly used to reduce Hg(ii) concentration in water.^5–7^ Among these methods, adsorption technology is widely acknowledged as one of the most promising methods for mercury removal in terms of its low cost, easy operation and design simplicity. With the rapid development of adsorbents, conventional adsorption materials, such as metal organic framework, carbon nanotube, layered double hydroxides, zeolite and clays,^8–12^ have been widely applied to remove heavy metals. Mesoporous materials are used as new adsorbents to remove organic pollutants and heavy metals in water owing to their large BET surface area, pore size, pore volume, regular pore structure and good hydrothermal stability.^13^

However, based on the previous reports, mesoporous materials are mainly prepared using tetraethylorthosilicate (TEOS) as silica source.^14,15^ It is worth noting that TEOS as commercial reagents are expensive and toxic,^16^ and the commonly used TEOS contains only 28 wt% SiO_2_. Therefore, searching alternative materials is also one of the research directions of this material. Diatomite, as a natural clay mineral with rich source, easy availability, low price and rich in SiO_2_, can be used as a desired raw material to synthesize molecular sieves, such as MCM-41 and SBA-15.^17,18^ Owing to the limited hydroxyl functions on mesoporous molecular sieves, resulting in relatively low adsorption capacity for heavy metal ions, especially Hg(ii) with large atomic radius.^19^ Hence, to the best of our knowledge, surface modification of mesoporous molecular sieves is a greatly effective method to improve their adsorption performance. Furthermore, mesoporous molecular sieve can be used as a good carrier for adsorption and separation reaction owing to its high specific surface area and regular pore structure.

Based on the hard–soft acid–base (HSAB) theory, some conductive polymers that are rich in oxygen, nitrogen and sulphur heteroatoms, such as poly pyrrole and poly thiophene, display strong affinity to heavy metal ions.^20^ It is beneficial to the removal of heavy metal ions. Moreover, the copolymers of pyrrole (Py) and thiophene (Tp) can offer two kinds of heteroatoms, providing an opportunity for the polymer molecular sieve composites to be adsorbents with multiform functionalities. Therefore, the mesoporous material MCM-41 is modified by copolymer. In organic functionalized mesoporous materials, inorganic components guarantee the basic structure and stability of the material, while organic group components give the material surface unique functions.^21^ Hence, it is greatly significant to explore a valuable technology to obtain high-performance adsorbents *via* the combination of mesoporous molecular sieves and di-heteroatom polymers.

In the present work, an advanced adsorbent MCM-41/co-(PPy-Tp) was constructed with the copolymer of co-(PPy-Tp) through copolymerizing pyrrole monomer (Py) and thiophene (Tp) onto the MCM-41 and used to remove the mercury ions. Furthermore, reaction conditions were optimized with response surface methodology (RSM) method. The kinetics, isotherms, thermodynamics, regeneration performance and adsorption mechanism were deeply explored. Meanwhile, the as-prepared adsorbents were applied to separate Hg(ii) from an actual electroplating wastewater containing various heavy metal ions.

## 2. Materials and methods

### 2.1 Materials

Cetyltrimethyl ammonium bromide (CTAB), sodium dodecyl sulphate (SDS), ferric chloride (FeCl_3_), sodium hydroxide (NaOH), pyrrole monomer (Py), thiophene (Tp) and diatomite were purchased from Aladdin (China). Hydrogen peroxide (H_2_O_2_, 30 wt%) was purchased from Sinopharm Chemical Reagent Co., Ltd. All of the chemicals are analytical reagent.

### 2.2 Synthesis of purified diatomite (DMTs)

Typically, 10.00 g diatomite and 100 mL of 5 M hydrochloric acid (HCl) were together mixed and magnetically stirred for 4 h at 378 K. Diatomite was separated through centrifugation and then washed with deionized water until the pH was close to neutral. The acidified diatomite (DMTs) was dried overnight at 333 K and then stored.

### 2.3 Preparation of mesoporous silica (MCM-41)

0.90 g of the as-prepared DMTs was poured in a flask containing sodium hydroxide (0.34 g) and deionized water (8.16 mL) at room temperature, and then the mixture was sonicated for 10 min. Afterwards, a certain amount of deionized water (17.00 g) containing CTAB (1.02 g) was added. Sulphur acid (H_2_SO_4_, 2 M) was used to adjust the solution pH to 10. The solution was continued to be stirred for 0.5 h. After that, the mixture was shifted to a 50 mL autoclave and kept at 373 K for 12 h and then was filtered, rinsed with deionized water and desiccated overnight at 378 K. Finally, the surfactant was purified by calcining the above materials in a muffle furnace at 823 K with a heating rate of 5 °C min^−1^ for 6 h.

### 2.4 Fabrication of MCM-41/co-Ppy-TP

For the synthesis of MCM-41/co-(PPy-Tp), 0.60 g MCM-41 powders, 60 mL of 0.05 M HCl solution, and 30.00 mg SDS were added into a round-bottomed flask with a capacity of 250 mL. SDS acts as an activator in the synthesis to activate the groups on the molecular sieve. The mixture was sonicated at room temperature for 15 min, and then stirred in a nitrogen atmosphere for 30 min. Then 0.5 mL pyrrole and 0.58 mL thiophene were added followed by magnetically agitation for 1 h. Subsequently, 15 mL of 0.48 M FeCl_3_ solution was slowly supplemented followed by dropwise addition of 10 mL H_2_O_2_ (addition rate: 30 min for each drop). Both FeCl_3_ and H_2_O_2_ were used as oxidants in the synthesis.

**Fig. 1:**
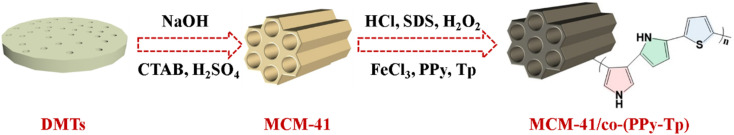
Schematic pathway of MCM-41/co-(PPy-Tp).

After 2 h of polymerization reaction at room temperature, 10 mL of H_2_O_2_ was continued to be supplemented. The reaction was conducted at room temperature for 4 h at N_2_ atmosphere and magnetic agitation. Eventually, the expected material was separated, rinsed, desiccated at 333 K. The synthesized schematic of MCM-41/co-(PPy-Tp) was presented in Fig. 1.

### 2.5 Batch adsorption experiments

Adsorption tests were performed to research the influences of pH, reaction time (adsorption kinetics), initial concentration (adsorption isotherm), temperature (thermodynamics) and dosage on the capacity of MCM-41/co-(PPy-Tp) to Hg(ii). For this purpose, a certain amount of MCM-41/co-(PPy-Tp) was added to an Erlenmeyer flask containing 100 mL of Hg(ii) solution. The solution pH was adjusted with 0.1 M HCl and 0.1 M NaOH, and oscillated at a speed of 160 rpm. The solution was filtered with a 0.45 μm filter and analyzed with a cold atomic absorption spectrophotometry after reaction 9 h.^22^ All tests were carried out three times, and the average data of the tests were recorded.^23^

### 2.6 Characterizations

The obtained samples were characterized *via* Scanning Electron Microscopy (SEM, Helios nanolab600i, FEI, USA), Transmission Electron Microscope (TEM, JEM-2100, JEOL, Japan), X-ray Diffraction (XRD, D8 Advance, Bruker, Germany), Fourier Transform Infrared (FT-IR, Nicolet 6700, Thermo Scientific, USA), X-ray Photoelectron Spectroscopy (XPS, Escalab250xi, Thermo Scientific, USA) and N_2_ adsorption–desorption instrument (Autosorb-IQ2-MP, Boynton Beach, USA). Zeta potential analyzer (Zeta PALS, Brookhaven, USA) was employed to confirm the charge property on the surface of materials.

## 3. Results and discussion

### 3.1 Characterizations

#### 3.1.1 SEM and TEM

**Fig. 2:**
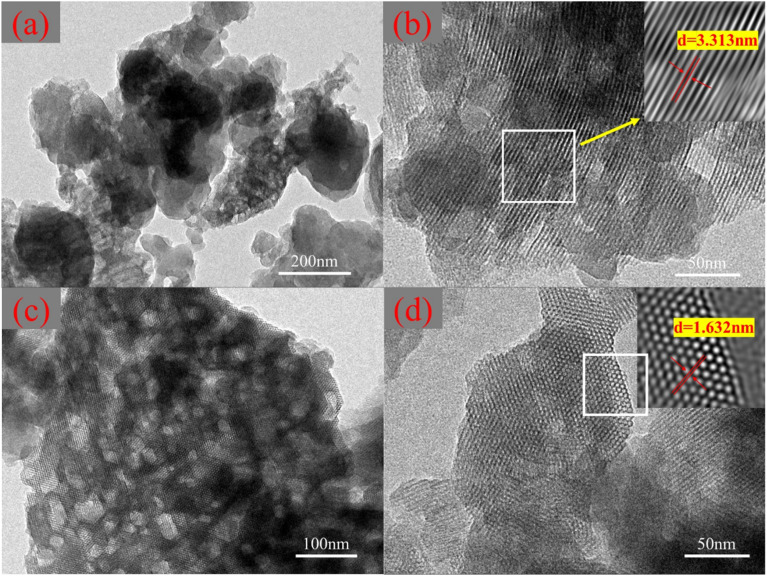
TEM images of MCM-41/co-(PPy-Tp).

SEM images of DMTs, MCM-41 and MCM-41/co-(PPy-Tp) are shown in Fig. S1 (in the ESI†). TEM is the most intuitive means to characterize the pore structure of mesoporous materials.^24^ TEM micrographs of MCM-41/co-(PPy-Tp) are presented in Fig. 2. From Fig. S1(a),† the surface of acidified diatomite is smooth and tidy with uniform pore structure.^25^ After CTAB modification and calcination, the acidic diatomite was transformed into a curved sheet-like mesoporous molecular sieve MCM-41 (Fig. S1(b)†) with a huge specific surface area (BET, 779.6 m^2^ g^−1^).^26^

As exhibited in Fig. S1(c),† after polymerization and modification with pyrrole and thiophene, the surface of MCM-41 is covered with a layer of dense flocculent particles. Fig. 2(a) and (c) are TEM images of MCM-41/co-(PPy-Tp). It is obvious that the smaller size MCM-41 after ultrasonic peeling is covered by a dense polymer layer, which proves that the polymers of pyrrole and thiophene were successfully loaded on the surface of MCM-41. In Fig. 2(b) and (d), it can clearly see the regular stripes of MCM-41/co-(PPy-Tp), which proves that the as-prepared sample has a relatively regular structure and a long-range ordered structure with one-dimensional channels.^17^ Moreover, the pore size distribution of the as-prepared material is uniform and large, about 1.5–4 nm, which is consistent with results of the N_2_ physical adsorption and desorption test.

#### 3.1.2 XRD study

**Fig. 3:**
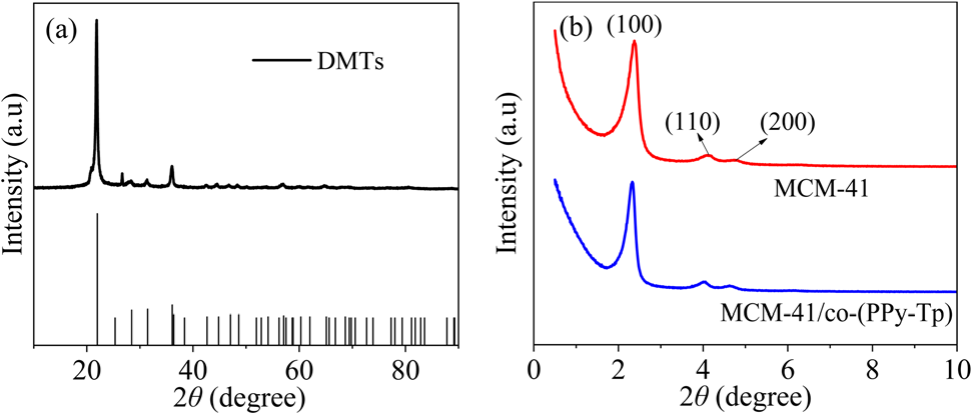
XRD images of DMTs, MCM-41 and MCM-41/co-(PPy-Tp).

Fig. 3(a) and (b) depict the XRD analysis of DMTs, MCM-41 and MCM-41/co-(PPy-Tp). From Fig. 3(a), it can be seen that DMTs has an obvious broad peak at 20–30°.^27^ In addition, there is a sharp peak at 2*θ* of 26.6°, indicating the presence of a small amount of crystalline silica.^28^ In Fig. 3(b), three characteristic peaks in MCM-41 at 2.4°, 4.3° and 4.8° can be found and belong to (100), (110) and (200) reflections, which are the diffraction peaks of typical MCM-41.^29^

Moreover, the peak of MCM-41/co-(PPy-Tp) still exist in view of the amorphous nature of conducting polymers compared with MCM-41, indicating that the polymerization reaction did not change the phase composition.

#### 3.1.3 FT-IR study

**Fig. 4:**
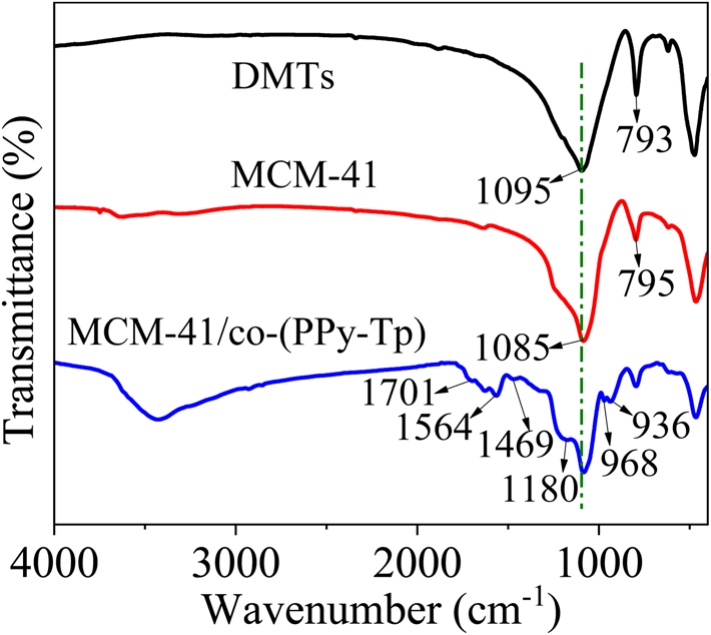
FT-IR images of DMTs, MCM-41 and MCM-41/co-(PPy-Tp).

Fig. 4 shows the FT-IR images of DMTs, MCM-41 and MCM-41/co-(PPy-Tp). In the case of DMTs, the characteristic peaks at 1095 cm^−1^ and 793 cm^−1^ are related to the stretching vibration of Si–OH.^30^ Compared with DMTs, the positions of several main characteristic peaks of mesoporous molecular sieve MCM-41 have not been changed significantly.

For the MCM-41/co-(PPy-Tp), the peaks at around 1701 cm^−1^ and 1180 cm^−1^ are attributed to the C=O and C–OH bonds, indicating the existence of carbonyls and hydroxyls. Moreover, the four characteristic peaks at 1564 cm^−1^, 1469 cm^−1^, 968 cm^−1^ and 936 cm^−1^ can be assigned to C–S and C–N bending vibration of the co-(PPy-Tp) (copolymer of pyrrole monomer (Py) and thiophene (Tp)). All of the results confirm that pyrrole and thiophene were successfully introduced onto the surface of MCM-41.

#### 3.1.4 BET study

**Fig. 5:**
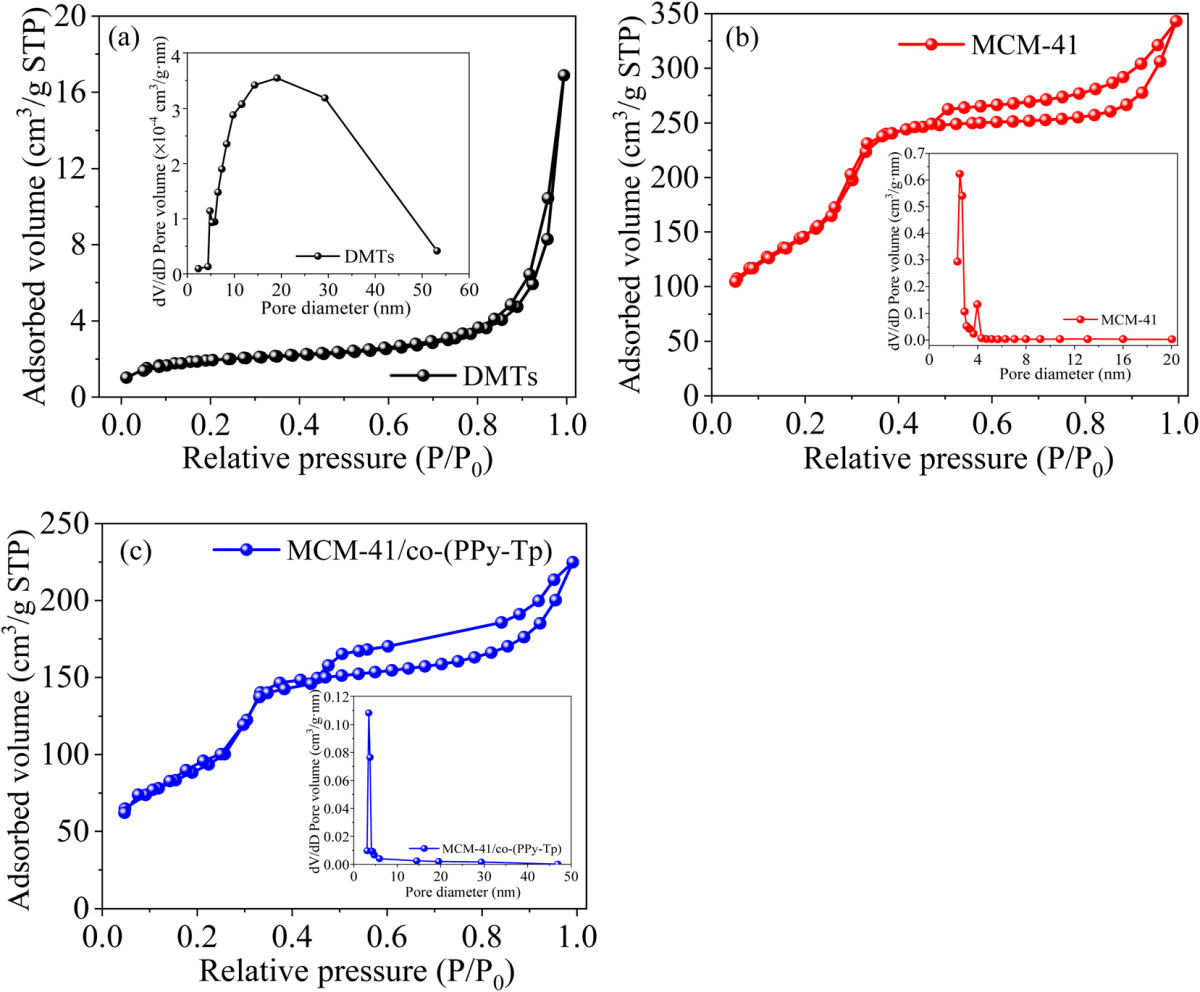
Adsorption isotherms of DMTs (a), MCM-41 (b) and MCM-41/co-(PPy-Tp) (c).

Generally, the adsorption capacity of an adsorbent is related to the surface functionality and structural parameters.^31,32^ The typical nitrogen adsorption–desorption isotherms of three materials are presented in Fig. 5.

It is observed that the as-prepared materials can be classified as type II, and their pore size distributions are 3–10 nm based on the Barret–Joyner–Halenda (BJH) model (Table 1) and the definition from International Union of Pure and Applied Chemistry (IUPAC). Hence, the synthesized three materials are mesoporous. However, MCM-41 prepared with purified diatomite exhibits a typical type IV. The isotherm adsorption can be divided into three steps: in the first step, N_2_ absorption increases at low *P*/*P*_0_ due to monolayer formation. After monolayer formation, capillary condensation leads to a sharp increase in N_2_ absorption. At high *P*/*P*_0_, there is a clear hysteresis ring, indicating the presence of mesosphere.^33^

**Table:** Isothermal data of as-prepared materials

Adsorbents	BET (m^2^ g^−1^)	Pore volume (cm^3^ g^−1^)	Pore size (nm)
DMTs	81.36	0.027	7.01
MCM-41	779.60	0.455	3.14
MCM-41/co-(PPy-Tp)	328.35	0.171	6.36

After the modification and grafting, MCM-41/co-(PPy-Tp) has a lower hysteresis loops. Moreover, the surface areas and pore volume are obviously decreased as the mesoporous material is constant modified, grafted and cross-linked with pyrrole and thiophene. The reason is that the cross-linked process of diatomite based mesoporous molecular sieve MCM-41 produces new macropores, leading to a sudden increase in the pore size of MCM-41/co-(PPy-Tp). The result is consistent with the data of N_2_ adsorption–desorption isotherm.

#### 3.1.5 XPS study

**Fig. 6:**
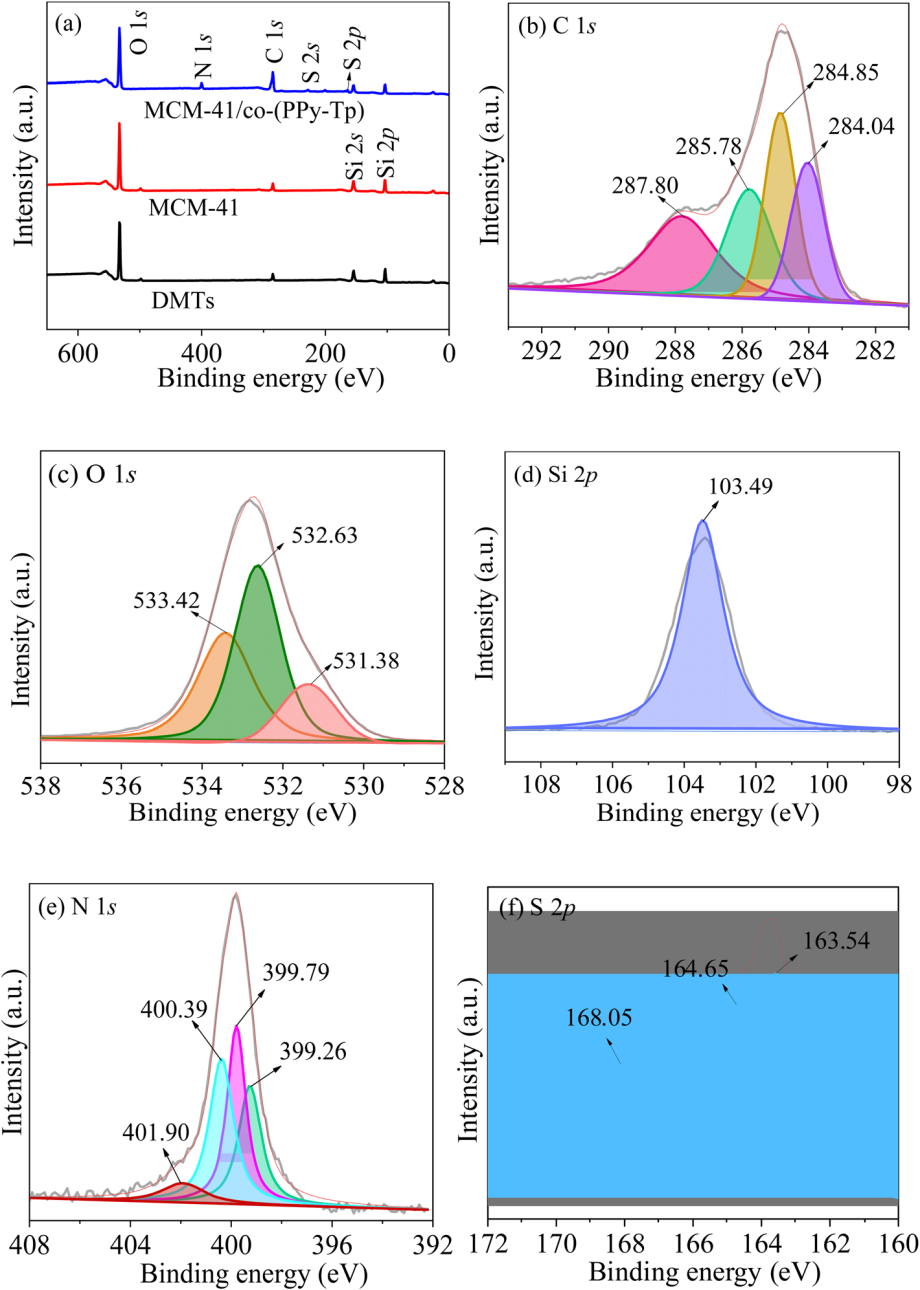
XPS survey scan (a); high-resolution scan of C 1s (b), O 1s (c), Si 2p (d), N 1s (e), and S 2p (f) of MCM-41/co-(PPy-Tp).

To further analyze the surface properties and investigate the valence states of surface elements of MCM-41/co-(PPy-Tp) composite, XPS spectra were utilized and depicted in Fig. 6. From Fig. 6(a), the appearance of N 1s, S 2s and S 2p peaks reflect that pyrrole and thiophene were successfully introduced onto the surface of MCM-41.

Additionally, the increase of the C 1s peak shows that the surface of as-synthesized materials is surrounded by cross-linked polymers of pyrrole and thiophene. Combined with the EDS results shown in Fig. S2,† the successful introduction of polymers on MCM-41 was proved.

Fig. 6(b) exhibits four major peaks at around 287.8 eV, 285.78 eV, 284.85 eV, and 284.04 eV, attributed to C–O, C–S, C=C and N–C=O bonds, severally. Based on the O 1s spectra in Fig. 6(c), the peaks at 531.38 eV, 532.42 eV and 533.4 eV are related to Fe–O, S=O/C–O and Si–O, severally.

Combined with the binding energy of O 1s, it can be known that the binding energy of Si 2p at 103.49 eV in Fig. 6(d) is attributed to Si–O in the MCM-41. As noted in Fig. 6(e), the N 1s component peaks at 399.26 eV, 399.79 eV, 400.39 eV and 401.9 eV are attributed to C=N, N–H, C–N^+^ polaron and C=N^+^ bipolaron, respectively. In Fig. 6(f), the S 2p spectrum contain three peaks at 163.54, 164.65 and 168.05 eV: the former two can be attributed to S 2p_3/2_ and S 2p_1/2_ of thiophene ring; the latter is attributed to oxysulfide, which is responsible for the shifting of S 2p_1/2_ to a high position.

#### 3.1.6 Zeta potential study

**Fig. 7:**
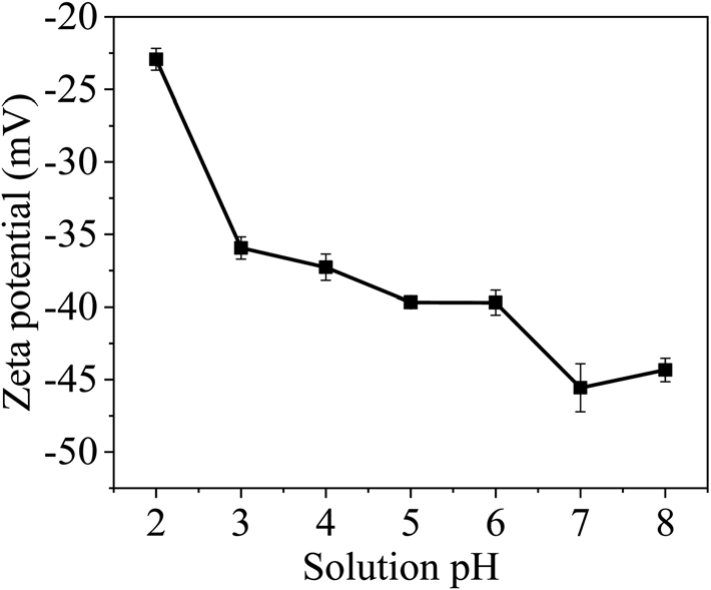
Changes of zeta potential of MCM-41/co-(PPy-Tp).

Zeta potential is often used to evaluate the influence of solution pH over the surface change.^34^Fig. 7 shows the zeta potential of MCM-41/co-(PPy-Tp) under various pH values. It is not difficult to find that the zeta potential values of the MCM-41/co-(PPy-Tp) are negative at pH of 2–8. The negative charges on the material surface are resulted from the dissociation of functional groups on the particle surface. Although the H^+^ concentration is high at low pH, the measurement result shows that the material surface is negatively charged. The result indicates that the material surface does not being completely protonated at the low pH. With the increase of solution pH, the zeta potential of the material decreases gradually due to deprotonation, resulted in the increasing amount of negative charges on the material surface.

Hence, it can be concluded that the surface of MCM-41/co-(PPy-Tp) is very favourable for the removal of Hg(ii) ions. In addition, the electronegativity on the surface of MCM-41/co-(PPy-Tp) continues to increase with the increasing pH. Under the action of electrostatic attraction, the adsorbent has further affinity to Hg(ii).

### 3.2 Adsorption Performance

#### 3.2.1 Effect of pH

**Fig. 8:**
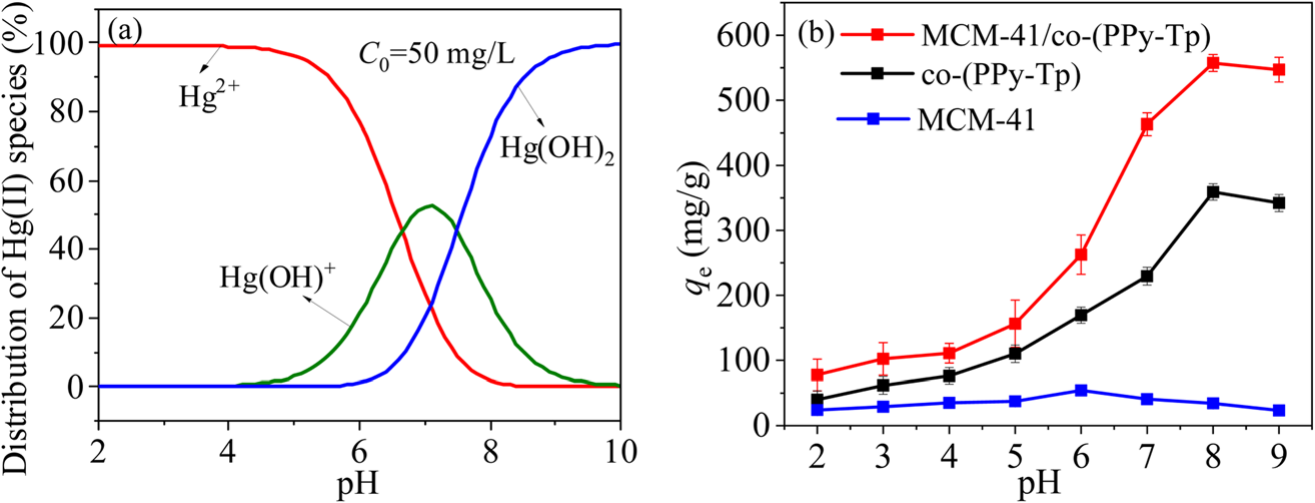
Morphologies of Hg(ii) at various pH (a) and pH effects over Hg(ii) removal with MCM-41, co-(PPy-Tp) and MCM-41/co-(PPy-Tp) (b) (dosage = 0.05 g L^−1^, *C*_0_ = 50 mg L^−1^, *t* = 9 h, *T* = 298 K).

The morphologies of Hg(ii) at various pH varying of 2–10 are simulated with Visual MINTEQ 3.0 and the simulated results are represented in Fig. 8(a).^35^ From Fig. 8(a), it can be seen that Hg(ii) has various existence morphologies at different pH conditions. If the solution pH is below 5, the main morphology of Hg(ii) in water is Hg(ii). However, the amount of Hg(ii) substantially decrease with the increasing pH due to the processes of hydrolysis and precipitation. Hence, solution pH is a crucial parameter for the removal of Hg(ii).

Fig. 8(b) presents the results of pH on the removal of Hg(ii) by MCM-41, co-(PPy-Tp) and MCM-41/co-(PPy-Tp). It can be seen that the MCM-41 material has almost no mercury absorption properties and the polymer of co-(PPy-Tp) has a high adsorption capacity of 229.4 mg g^−1^. After copolymerizing pyrrole monomer (Py) and thiophene (Tp) onto the MCM-41, the adsorption capacity of MCM-41/co-(PPy-Tp) towards Hg(ii) is significantly increased in the optimal pH range. Simultaneously, it also can find that adsorption capacity of Hg(ii) is the lowest at pH = 2 due to the H^+^ competition for adsorption sites.^36,37^

However, as the solution pH increases continuously, the concentration of H^+^ ions in the solution gradually decreases, and the H^+^ in the –NH– group in the polymer molecular chain is dissociated. Meanwhile, the Hg(ii) ions in the solution is exactly shared with the N atom in the –N=C– group lone pair of electrons, thereby forming a new stable complex with Hg(ii) ions. Ultimately, a highly effective removal of Hg(ii) ions is achieved and the optimal adsorption capacities of MCM-41/co-(PPy-Tp) for Hg(ii) achieve 463.11 mg g^−1^ at pH = 7.

#### 3.2.2 RSM design

To optimize the conditions and factors of the experiments, the inherent relationship among the adsorption capacity and the four factors of pH (A), temperature *T* (B), initial concentration of *C*_0_ (C) and dosage (*D*) was in-depth investigated. Design-Expert 11 was applied to a Central Composite Design (CCD) matrix under the conditions of pH = 5–9, *T* = 25 °C–45 °C, *C*_0_ = 20–60 mg L^−1^ and dosage of 0.040.08 g L^−1^. The test was implemented with 29 runs and the CCD results of MCM-41/co-(PPy-Tp) is listed in Table S1 (in the ESI†).

The fitting parameters of Linear, Two Factor Interaction (2FI), Quadratic and Cubic modes, are listed in Table 2. Based on the results, the Quadratic model has a relatively high coefficient (*R*^2^ = 0.9995) and the smallest difference between the adjusted coefficient of determination (*R*^2^ = 0.9990) and predicted coefficient (*R*^2^ = 0.9972) among the four models, demonstrating that the Quadratic model is more appropriate to characterize Hg(ii) adsorption.

**Table:** Fitting results of four models

Source	Std. dev	*R* ^2^	Adjusted *R*^2^	Predicted *R*^2^	Press	
Linear	49.45	0.6452	0.5884	0.5290	81 150	
2FI	50.73	0.7162	0.5669	0.5260	81 674	
Quadratic	2.50	0.9995	0.9990	0.9972	474	Suggested
Cubic	1.85	0.9999	0.9994	0.9923	1329	Aliased

ANOVA data^38^ was used to reflect the correlation degree among the independent variables and *q*_e_ and is expressed in quadratic form, as follows:

1 *q*_e_ = 469.67 + 63.96 × *A* + 13.54 × *B* + 17.62 × *C* + 7.46 × *D* + 15.31 × *AB* + 16.81 × *AC* − 10.56 × *AD* − 6.94 × *BC* − 9.31 × *BD* − 1.06 × *CD* − 37.07 × *A*^2^ − 9.95 × *B*^2^ − 20.32 × *C*^2^ − 18.70 × *D*^2^

2 *q*_e_ = −3323.83 + 471.9167 × pH + 25.85 × *T* + 11.74583 × *C*_0_ + 37520.93 Dosage + 3.0625 × pH × *T* + 1.68125 × pH × *C*_0_ − 1056.25 × pH × Dosage − 0.13875 × *T* × *C*_0_−186.25 × *T* × Dosage − 10.625 × *C*_0_ × Dosage − 37.07292 × pH^2^ − 0.397917 × *T*^2^ −0.203229 × *C*^2^_0_ − 186979 × Dosage^2^

**Fig. 9:**
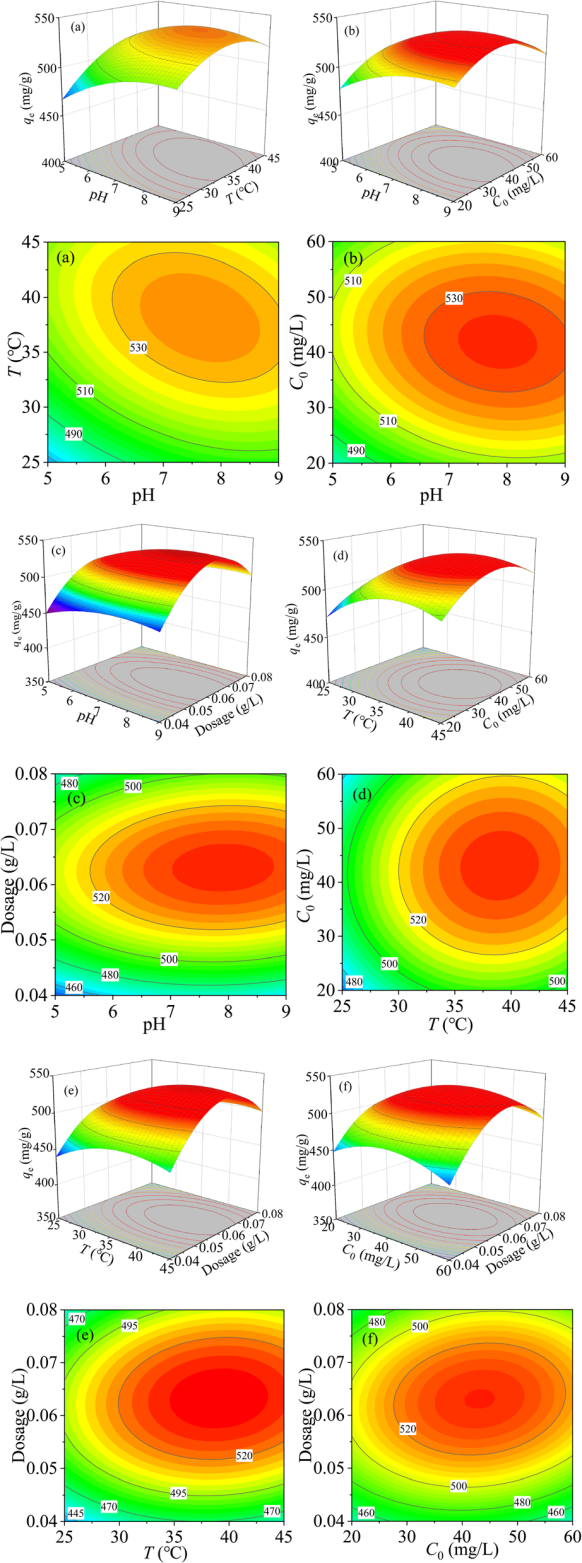
3D contour between the variable and *q*_e_: pH ∼ *T* (a); pH ∼ *C*_0_ (b); pH ∼ dosage (c); *T* ∼ *C*_0_ (d); *T*∼ dosage (e); *C*_0_ ∼ dosage (f).

Fig. 9(a) is the 3D contour map between pH and temperature at *C*_0_ = 40 mg L^−1^ and dosage of 0.06 g L^−1^. It can be seen that when pH is in the range of 7.0–8.5, and the temperature is changed from 35 °C to 45 °C, the adsorption performance of MCM-41/co-(PPy-Tp) gets better. Conversely, when the pH of the solution is less than 6.5 and the solution temperature is less than 30 °C, the adsorption capacity of MCM-41/co-(PPy-Tp) is relatively small. The results can be assigned to the fact that when the solution pH is low, the high level of H^+^ concentration in the solution competes with Hg(ii) ions for adsorption sites.

Meanwhile, the influence of temperature on the adsorption capacity is far less than that of solution pH. Moreover, it is not difficult to find that the *F* value of model *AB* (pH ∼ *T*) is 669.3 from Table S2,† and the *P*-value is less than 0.0001, indicating that the influence of solution pH and temperature over the removal of Hg(ii) is both remarkable.

The combined effect of model *AC* (pH ∼ *C*_0_) on *q*_e_ was investigated at *T* = 35 °C and dosage of 0.06 g L^−1^. In Fig. 9(b), when solution pH is 7–9 and *C*_0_ is 37–47 mg L^−1^, MCM-41/co-(PPy-Tp) exhibits the best adsorption performance. The reason is that the adsorption capacity of MCM-41/co-(PPy-Tp) can reach saturation when mercury ions concentration reaches a certain amount. In addition, the *F*-value in model *AC* is 806.8, and the *P*-value in Table S2† is less than 0.0001, indicating that model *AC* has a considerable effect over the removal of Hg(ii).

As illustrated in Fig. 9(c), the interaction of model *AD* (pH ∼ dosage) towards *q*_e_ at *T* = 35 °C and *C*_0_ = 40 mg L^−1^. The value of *q*_e_ is relatively high at pH of 7.0–8.5. The results indicate that the *q*_e_ of MCM-41/co-(PPy-Tp) enlarges with the increase of dosage, owing to the increased utilizability of active sites. In addition, the *q*_e_ value is almost unchanged. Besides, both the high *F*-value (318.44) and low *P*-value (<0.0001) exhibit that the term *AD* is also momentous, and the results are presented in Table S2.†

The combined effect of model *BC* (*T* ∼ *C*_0_) on the *q*_e_ at pH = 7 and dosage = 0.06 g L^−1^ is displayed in Fig. 9(d). From the contour map, it is not difficult to find that the *q*_e_ of MCM-41/co-(PPy-Tp) for mercury ions also increases with the increasing temperature, which implies that the adsorption process is endothermic.

Fig. 9(e) expresses the joint effect of model *BD* (*T* ∼ dosage) on *q*_e_ at pH = 7 and *C*_0_ = 40 mg L^−1^. The findings reveal that low temperature and low dosage are not favourable for the removal of Hg(ii) ions. The F-value (247.53) and low *P*-value (<0.0001) indicate that the model *BD* is momentous, and the results are presented in Table S2.†

The dependence of *q*_e_ on the effect of model *CD* (*C*_0_ ∼ dosage) towards pH = 7 and *T* = 35 °C is shown in Fig. 9(f). When *C*_0_ is 35–53 mg L^−1^, and the adsorbent dosage is in the region of 0.058–0.07 g L^−1^, the performance of MCM-41/co-(PPy-Tp) is better. In Table S2,† both the *F*-value (3.22) and *P*-value (0.0928) suggest that the combination of model *CD* on the performance of the MCM-41/co-(PPy-Tp) is insignificant.

To summarize, MCM-41/co-(PPy-Tp) has an optimal adsorption capacity *q*_e_ of 537.15 mg g^−1^ at pH = 7.1, *T* = 37.9 °C, *C*_0_ = 45.1 mg L^−1^ and dosage of 0.064 g L^−1^.

### 3.3 Adsorption kinetics

**Fig. 10:**
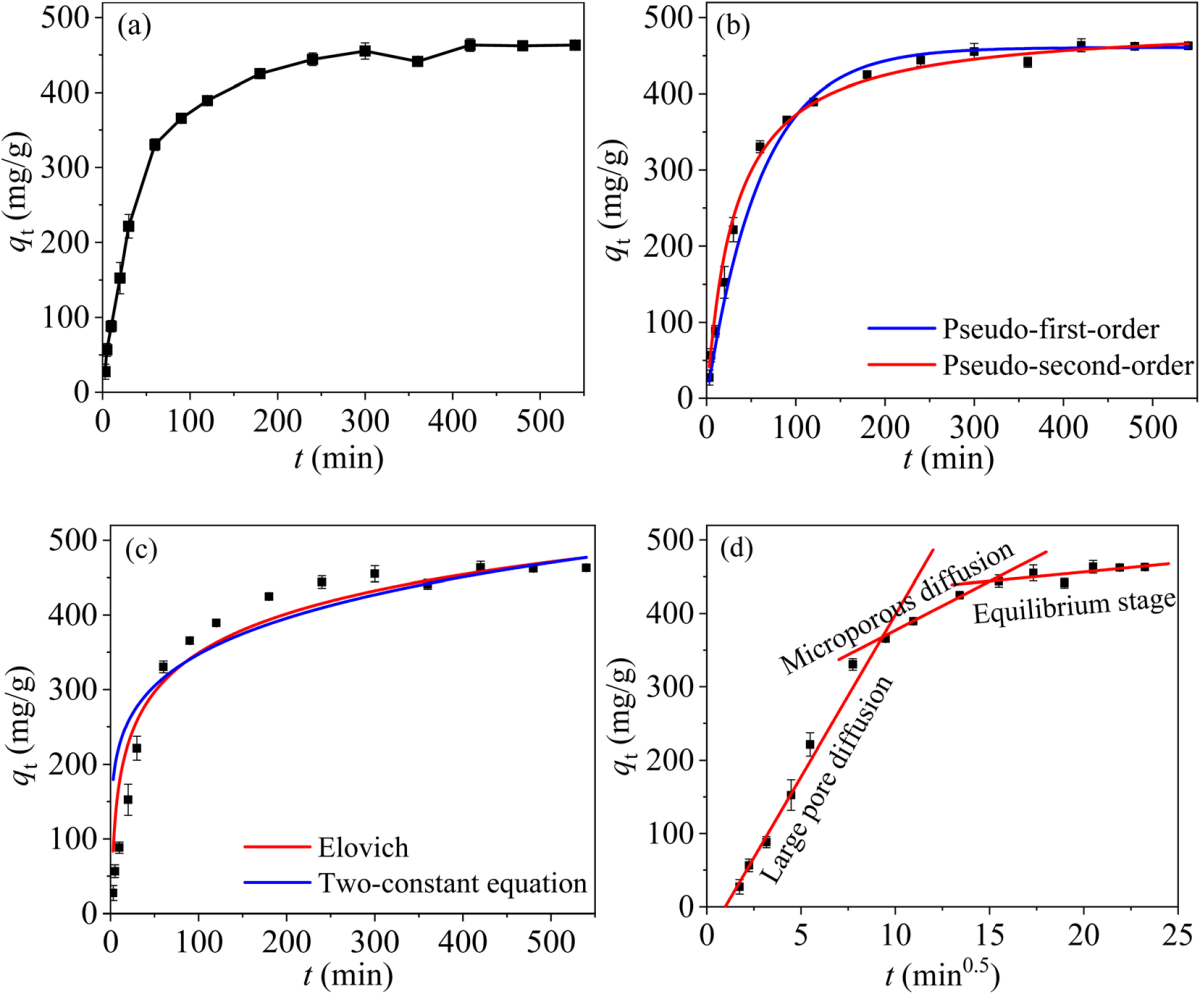
*q*_t_ *vs.* reaction time (a); PFO and PSO kinetics (b); elovich and TCE kinetics (c); IPD kinetics (d) (dosage = 0.05 g L^−1^, pH = 7, *C*_0_ = 50 mg L^−1^, *T* = 298 K).

Fig. 10(a) shows the relationship between reaction time and the removal of Hg(ii) by MCM-41/co-(PPy-Tp) under selected conditions. The data were fitted with nonlinear pseudo-first-order (PFO), pseudo-second-order (PSO), intra-particle diffusion (IPD), Elovich and two constant equation (TCE) models [eqn (S1)–(S5)†]. The corresponding results can be found in Fig. 10(b)–10(d) and Table 3.

Results of kinetic fitting

**Table:** 

PFO model					PSO model			
*q*_e,exp_ (mg g^−1^)	*q*_e,cal_ (mg g^−1^)	*K*_1_	*R* ^2^	SSE	*q*_e_ (mg g^−1^)	*K*_1_	*R* ^2^	SSE
463.11	460.64	0.016	0.9881	85.44	495.63	0.0001	0.9952	33.32

**Table:** 

IPD model								
*K*_d1_	C_1_	*R* ^2^	*K*_d2_	*C*_2_	*R* ^2^	*K*_d3_	*C*_3_	*R* ^2^
44.13	−43.14	0.9863	13.33	243.66	0.9802	2.48	406.8	0.8124

**Table:** 

Elovich model				TCE model			
*α*	*β*	*R* ^2^	SSE	*A*	*B*	*R* ^2^	SSE
0.012	0.013	0.915	584.08	145.99	0.189	0.814	1284

From Fig. 10(a), the adsorption is very fast in the initial 60 min, and the adsorption equilibrium with a maximum adsorption capacity of 330.5 mg g^−1^ is reached after about 300 min. MCM-41/co-(PPy-Tp) provides a large number of active sites during the initial adsorption stage, resulted in rapid electrostatic attraction and coordination chelation between divalent mercury and adsorbent. However, as the adsorption process goes on, the adsorption rate decreases slowly. The reason is that a large number of active sites are occupied. Hg(ii) ions slowly migrate to the surface of the mesoporous molecular sieve through intragranular diffusion, and then slowly adsorb with the O^−^ ionized by hydroxyl.

With the increase of adsorption time, both the concentration of divalent mercury in the solution and adsorption efficiency decreases continuously. Then the adsorption is slowly adsorbed until the adsorption equilibrium is reached at about 540 min. The obtained adsorption capacity is 463.11 mg g^−1^.

As illustrated in Fig. 10(b) and (c) and Table 3, the PSO model is more suitable to characterize the removal behaviour of Hg(ii) by MCM-41/co-(PPy-Tp) compared with other four adsorption kinetic models (PFO, Elovich and TCE), and the theoretical adsorption capacity of *q*_e_ is 495.63 mg g^−1^. The results demonstrate that the adsorption of mercury on MCM-41/co-(PPy-Tp) is a chemical process.

Fig. 10(d) is the fitting result of IPD model. The entire process includes three stages: (i) large pore diffusion: matching with rapid adsorption and large *K*_d1_ value; (ii) microporous diffusion: matching with medium adsorption and *K*_d2_ value; (iii) equilibrium adsorption: matching with slow adsorption and minimal *K*_d3_ value. Based on the values of *K*_di_ at each stage, the right conclusion is that the adsorption rate gradually decreases with the progress of reaction, suggesting that the adsorption behaviour is mainly controlled by the first two stages.

### 3.4 Adsorption isotherms

The relationship between the equilibrium concentration (*C*_e_, mg L^−1^) and the *q*_e_ can be characterized with essential isothermal models, including Langmuir, Freundlich, Temkin and Dubinin–Radushkevich (D–R) models [eqn (S6)–(S9)†].

**Fig. 11:**
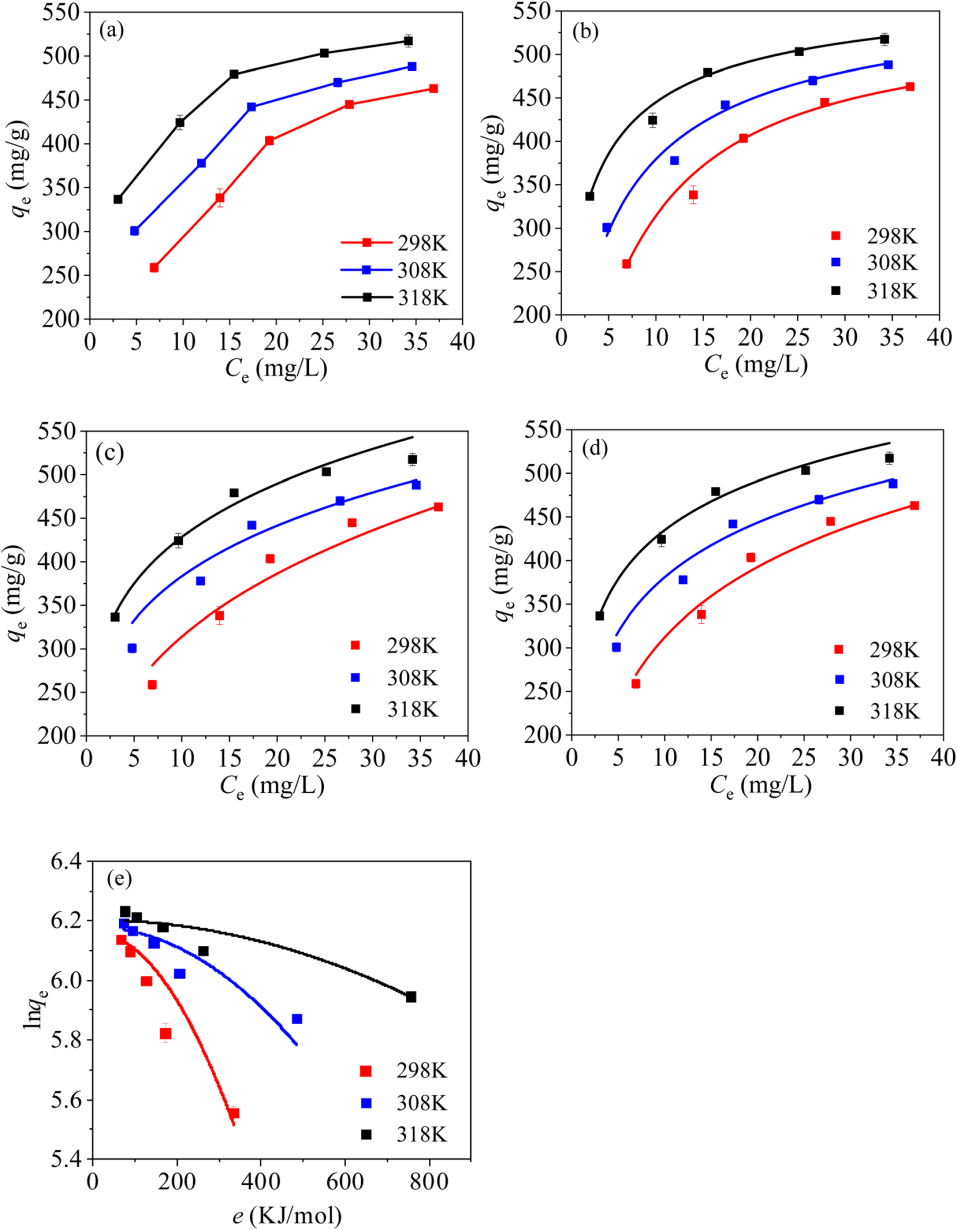
Isotherm curves of Hg(ii) on MCM-41/co-(PPy-Tp) (a), Langmuir (b), Freundlich (c), Temkin (d) and D–R models (e) (dosage = 0.05 g L^−1^, *t* = 9 h, pH = 7, *C*_0_ = 20–60 mg L^−1^).

From Fig. 11(a), the value of *q*_e_ increase with the increasing solution temperature, suggesting that increasing temperature favours the adsorption. From Fig. 11(b) and Table 4, it can be found that the *R*^2^ fitted with Langmuir model is the highest in the four kinetic models, illustrating that the adsorption process of Hg(ii) on MCM-41/co-(PPy-Tp) involves chemical adsorption (a single-layer adsorption), and the result is in accord with the kinetic data. Additionally, the maximum adsorption capacity (*Q*_m_) of MCM-41/co-(PPy-Tp) towards Hg(ii) reaches 533.57 mg g^−1^ at 298 K, which is close the optimized results (537.15 mg g^−1^) of RSM and higher than the adsorption capacities of other composites and adsorbents alone in the literature (Table 5).

Fitting results of isotherm models

**Table:** 

Langmuir					
*T* (K)	*Q*_m_ (mg g^−1^)	*K*_L_ (L mg^−1^)	*R* ^2^	SSE	RMSE
298	533.57	0.097	0.9922	7.444	1.220
308	577.26	0.263	0.9341	65.009	3.606
318	593.77	0.607	0.9943	6.053	1.100

**Table:** 

Freundlich					
T (K)	*K*_F_ (L^*n*^ mg^*n*−1^ g^−1^)	1/*n*_F_	*R* ^2^	SSE	RMSE
298	157.75	0.299	0.9468	70.892	3.765
308	239.48	0.204	0.8957	152.099	5.515
318	274.84	0.193	0.9752	39.54	2.812

**Table:** 

Temkin					
*T* (K)	*b*_T_	*K*_T_	*R* ^2^	SSE	RMSE
298	1.789	1.467	0.9749	33.632	2.594
308	3.216	6.733	0.9276	105.508	4.594
318	4.616	21.410	0.9881	18.641	1.931

**Table:** 

D–R					
*T* (K)	*Q*_m_ (mg g^−1^)	*E* (kJ mol^−1^)	*R* ^2^	SSE	RMSE
298	473.902	294.985	0.906	75.535	3.887
308	477.570	445.236	0.619	458.538	9.576
318	494.546	607.533	0.962	54.924	3.314

**Table:** Comparison of adsorption capacity for Hg(ii)

Adsorbents	BET (m^2^ g^−1^)	*T* (°C)	pH	*Q*_m_ (mg g^−1^)	Ref.
MCM-41	834.91	25	6	42	39
Chitosan	—	25	6	8.24	40
DMC	—	25	4	147.9	41
PAC	1786.9	25	7	105	42
GO	515.8	25	9	30	43
SBA-15	148.741	25	6	40.4	14
Zeolite	126.9	25	6	84.24	44
PAAM-NH_2_-MCM-41	646	25	5.2	177	13
Chitosan/MCM-41-PAA	253.31	25	4	164	45
G-DMC	—	25	4	443.8	41
NiFe_2_O_4_-PAC-SH	1700.4	25	7	298.8	42
MNP-CD-PBTCA	13.84	55	4	77.59	46
EDTA-mGO	49.97	—	4.1	268.4	47
CoFe_2_O_4_@mSiO_2_-NH_2_	17.08	25	7	149.3	35
Ppy-SBA-15	96.7	45	8	200	48
Fe_3_O_4_@SiO_2_@Se	—	25	3	70.42	49
APTMs-modified TO-NFC	129.32	50	3–7	242.1	50
Starch/SnO_2_	78.5	25	7	192	51
MCM-41/co-(PPy-Tp)	328.35	37.9	7.1	537.15	This work

From Fig. 11(c) and Table 4, it can be known that the value of 1/*n* in Freundlich model is less than 1, indicating a favourable adsorption. Meanwhile, the values of *R*^2^ and *K*_T_ in Temkin model (Fig. 11(d)) are high, revealing a strong interaction between MCM-41/co-(PPy-Tp) and Hg(ii) ions. And the result is in accord with the conclusion of the pH research. Increasing *b*_T_ value implies the increase of *q*_e_ with ascending temperature. The result also verifies that the higher the temperature, the more favourable the adsorption of Hg(ii), which verifies the correctness and rationality of RSM optimization.

In D–R model, average adsorption energy per mole (*E*, kJ mol^−1^) is an essential index to distinguish physical adsorption or chemical adsorption. All of the *E* values are significantly greater than 16 kJ mol^−1^, so the adsorption process is attributed to chemical adsorption.

### 3.5 Thermodynamics

Temperature is one of the most vital parameters for materials to adsorb heavy metals.^52^ The thermodynamic adsorption behaviours of MCM-41/co-(PPy-Tp) for Hg(ii) were investigated at 298 K, 308 K and 318 K. Thermodynamic parameters including standard enthalpy (Δ*H*^0^), standard entropy (Δ*S*^0^) and Gibbs free energy (Δ*G*^0^) were calculated with eqn (S10) and (S11),† and the calculated results are listed in Table 6.

**Table:** Adsorption thermodynamic parameters

*C*_0_ (mg L^−1^)	Δ*H*^0^ (kJ mol^−1^)	Δ*S*^0^ (J mol^−1^ K^−1^)	Δ*G*^0^ (kJ mol^−1^)		
			298 K	308 K	318 K
40	15.60	77.59	−7.52	−8.29	−9.07
50	8.80	52.54	−6.86	−7.38	−7.91
60	8.64	50.03	−6.27	−6.77	−7.27

**Fig. 12:**
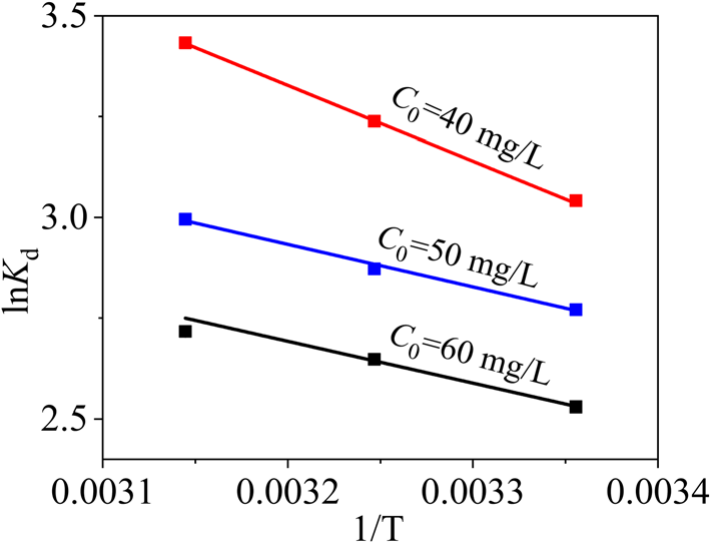
Thermodynamic results with MCM-41/co-(PPy-Tp) (dosage = 0.05 g L^−1^, pH = 7, *t* = 9 h, *C*_0_ = 40–60 mg L^−1^).

From Table 6 and Fig. 12, Δ*H*^0^ are all positive at three C_0_, implying that the adsorption reaction of Hg(ii) onto MCM-41/co-(PPy-Tp) is endothermic. The Δ*S*^0^ values are also positive, which implies the increase of randomness at the interface between Hg(ii) and the adsorbent surface. Meanwhile, as temperature rises, the Δ*G*^0^ value decreases and the spontaneity of the reaction increases, indicating that the rise in temperature promotes the progress of the reaction. In addition, combined with the values of average adsorption energy *E* and Gibbs free energy Δ*G*°, it can be concluded that the adsorption of Hg(ii) onto MCM-41/co-(PPy-Tp) belongs to the chemical adsorption category. Overall, the adsorption of MCM-41/co-(PPy-Tp) for Hg(ii) is a spontaneous endothermic reaction.

### 3.6 Reusability evaluation

The reusability is an important index to judge the performance of synthesized adsorbents.^53^ According to the research by Lawrence A^54^*et at.*, five eluents including 0.1 M HCl, 5 wt% EDTA, 5 wt% EDTA + 0.1 M HCl, 5 wt% thiourea and 5 wt% thiourea + 0.1 M HCl were adopted as the desorption solvent to recover Hg(ii) from the used adsorbents.

**Fig. 13:**
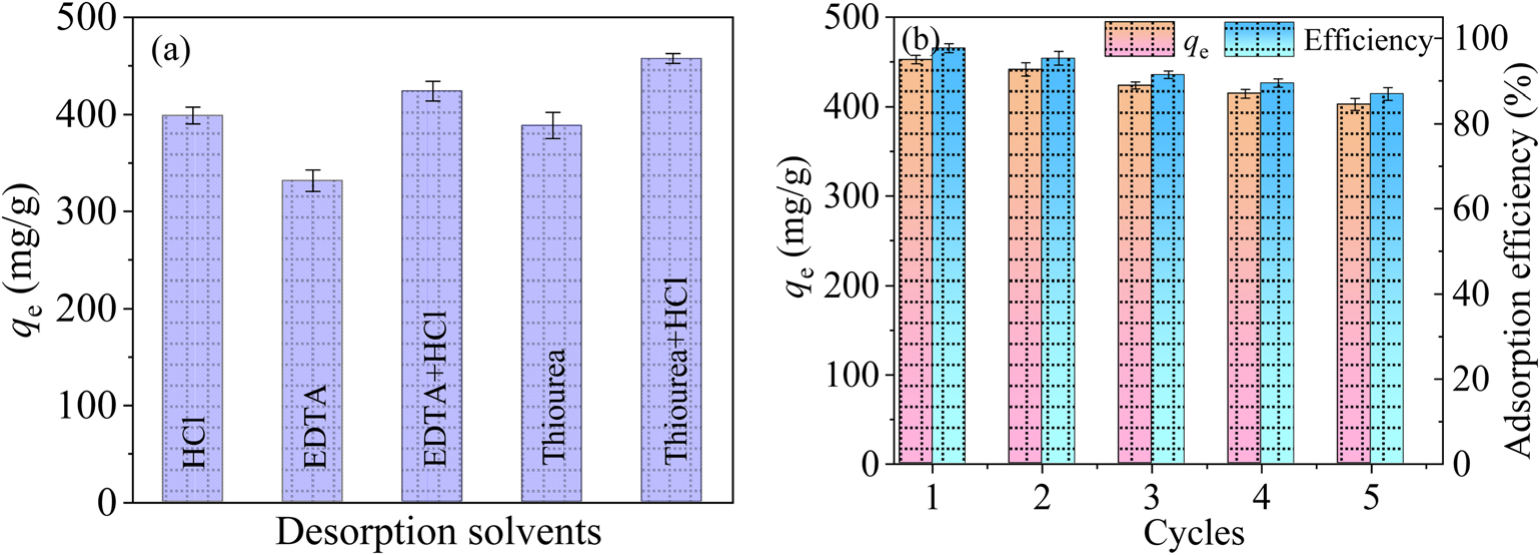
Influence of different desorbents on MCM-41/co-(PPy-Tp) (a), regeneration cycle of MCM-41/co-(PPy-Tp) (b) (dosage = 0.05 g L^−1^, pH = 7, *t* = 9 h, *C*_0_ = 50 mg L^−1^, *T* = 298 K).

From Fig. 13(a), the desorption effect of HCl, thiourea and EDTA for Hg(ii) are relatively poor, with the desorption rates of 86.15%, 83.93% and 71.61%, respectively. The maximum desorption capacity of EDTA + HCl is 423.8 mg g^−1^, indicating that there is an obvious complexation between the mixture of HCl + EDTA and Hg(ii). When the mixture of HCl and thiourea was used as the desorption agent, the maximum desorption rate can reach 98.76%. The reason is that the strong acid desorption solution can release large amounts of H^+^ that can compete with Hg(ii) for the adsorption sites on the adsorbent. Meanwhile, Hg(ii) can closely chelate with the thiourea, leading to a greatly increases of desorption.

As depicted in Fig. 13(b), five cycles of adsorption–desorption experiments of Hg(ii) on MCM-41/co-(PPy-Tp) were carried out. The Hg(ii) ions adsorption capacity using MCM-41/co-(PPy-Tp) still maintain over 86.97% in the fifth cycle. Taking into account the characteristics of high adsorption capacity and easy regeneration, MCM-41/co-(PPy-Tp) is expected to be an adsorbent with practical application prospects.

### 3.7 Application evaluation

To in depth explore the application possibility in the treatment of actual wastewater, the as-prepared MCM-41/co-(PPy-Tp) was applied to treat the actual pre-treated electroplating effluent. The wastewater sample was obtained from an electroplating enterprise in Kunshan city of Jiangsu Province.

**Fig. 14:**
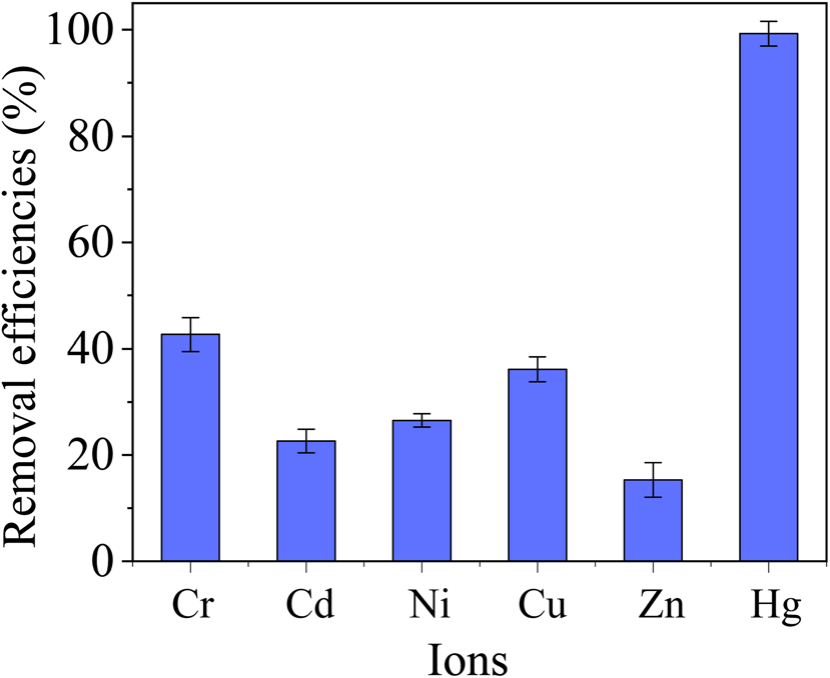
Application of MCM-41/co-(PPy-Tp) in actual electroplating wastewater (dosage = 0.05 g L^−1^, pH = 7.2, *t* = 12 h, *T* = 298 K, MCM-41/co-(PPy-Tp)).

The heavy metal ions in the employed electroplating wastewater mainly involve Cr(vi) (8.6 mg L^−1^), Cd(ii) (12.2 mg L^−1^), Ni(ii) (11.8 mg L^−1^), Cu(ii) (8.7 mg L^−1^), Zn(ii) (5.5 mg L^−1^) and Hg(ii) (7.3 mg L^−1^). The Total Oxygen Demand (TOD) is 55.1 mg L^−1^. The adopted dosage was 0.05 g L^−1^. And the removal results of Hg(ii) are presented in Fig. 14.

From Fig. 14, the removal efficiency of Hg(ii) achieves 99.3% and the content of Hg(ii) after treatment with MCM-41/co-(PPy-Tp) is below 0.1 mg L^−1^, which is absolutely met the criterion of “Emission Standard of Pollutants for Electroplating” (GB 21900-2008). The result indicates that the diatomite-based mesoporous materials of MCM-41/co-(PPy-Tp) has highly selective adsorption for Hg(ii) in the actual application and is a quite promising adsorbent.

### 3.8 Mechanism speculation

**Fig. 15:**
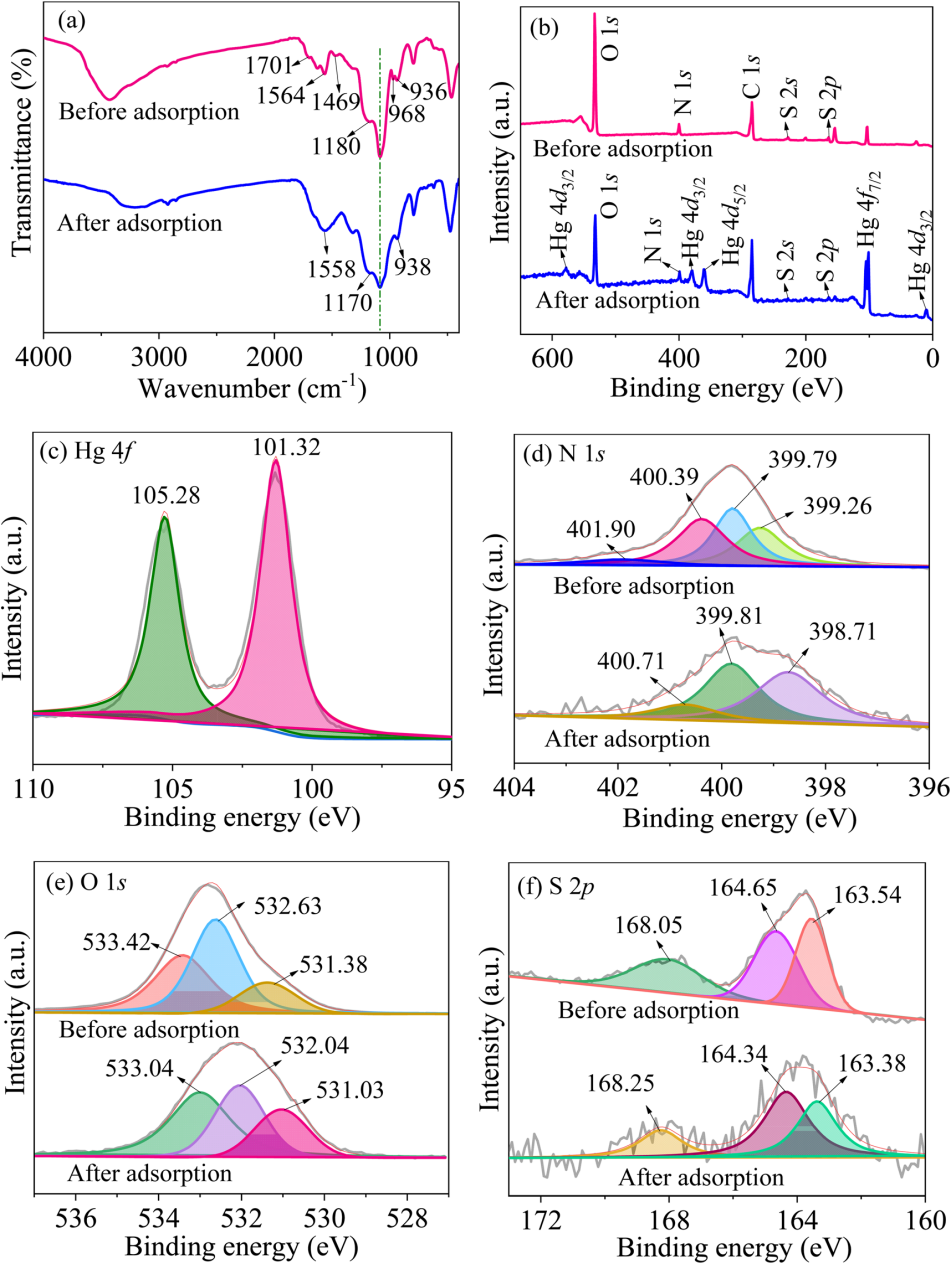
FT-IR (a) and XPS survey scan (b); high-resolution scan of Hg 4f (c), N 1s (d), O 1s (e) and S 2p (f) of MCM-41/co-(PPy-Tp) before and after adsorption.

According to the results of kinetics and isotherms, the efficient adsorption of mercury ions on MCM-41/co-(PPy-Tp) belongs to chemical adsorption and monolayer adsorption. As depicted in Fig. 15(a), after adsorbing mercury ions, the characteristic peaks of C=O, C=N and N–H at 1701 cm^−1^, 1469 cm^−1^ and 968 cm^−1^ almost disappear and meanwhile the characteristic peak of C–S is shifted to some extent. The fact implies that Hg(ii) has a strong interaction with the above chemical groups.

Fig. 15(b) is the XPS survey of the adsorbent before and after adsorption. Obviously, the peaks of O 1s, N 1s and S 2p are all weakened and some new peaks such as Hg 4f_5/2_ (105.28 eV) and Hg 4f_7/2_ (101.3 eV) (Fig. 15(c)) appear after adsorption, demonstrating that Hg(ii) is successfully adsorbed onto MCM-41/co-(PPy-Tp).

Fig. 15(d) is the high-resolution N 1s spectrum. The peak of C=N^+^ bipolaron almost disappear and the characteristic peaks of C=N, N–H and C–N^+^ polaron are all shifted, indicating a strong interaction between N atom and Hg(ii).

The deconvolution of O 1s of MCM-41/co-(PPy-Tp) and MCM-41/co-(PPy-Tp)/Hg(ii) are presented in Fig. 15(e). Compared with MCM-41/co-(PPy-Tp), the corresponding peaks of O 1s shift to a low energy position after adsorption, suggesting a forceful interaction between Hg(ii) and oxygen atoms.

Fig. 15(f) presents the deconvolution of S 2p spectra before and after adsorption. The peak in C–S bonds (168.25 eV) shifts to a higher binding energy position, implying that a part of C–S bonds transforms to C–S–Hg; The other two binding energies (164.34 eV and 163.38 eV) shift to lower binding energy in comparison with before adsorption, indicating that S atoms participate in the chelation process with Hg(ii).

In view of the XPS results, all of the three principal heteroatoms (N, O and S) in MCM-41/co-(PPy-Tp) may be involved in the adsorption of Hg(ii). In addition, the MCM-41/co-(PPy-Tp) has excellent stabilization based on the XPS and FT-IR data before and after adsorption.

**Fig. 16:**
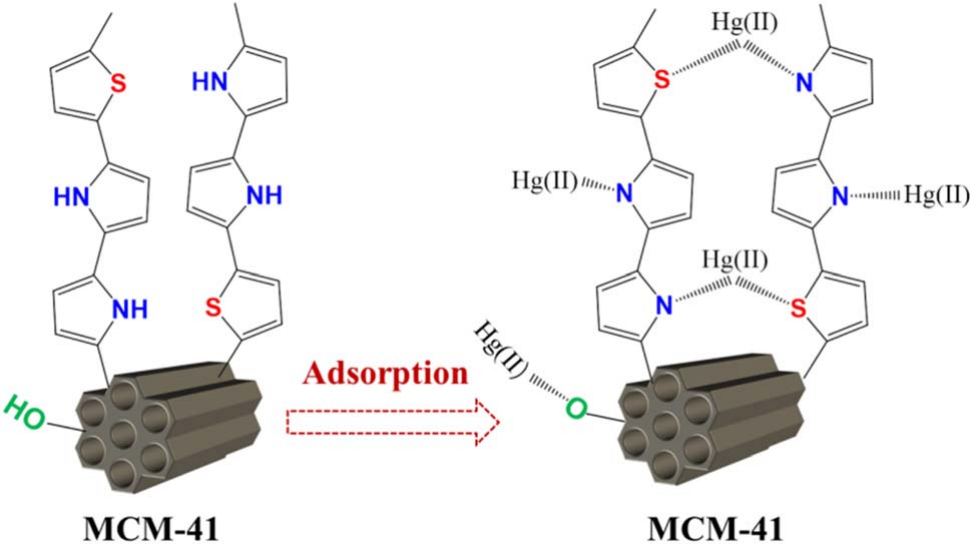
Schematic diagram of Hg(ii) adsorption mechanism with MCM-41/co-(PPy-Tp).

Taking into account the characterization analysis above, the possible mechanism of Hg(ii) adsorption onto MCM-41/co-(PPy-Tp) can be attributed to several multi-interactions, as illustrated in Fig. 16.

In acidic and neutral solutions, mercury ions mainly exist in the form of Hg^2+^ and Hg(OH)^+^, and they can form stable complexes through complexation with N or S atoms in MCM-41/co-(PPy-Tp) to achieve adsorption. As the active sites are slowly captured, mercury ions migrate into the surface of diatomite-based mesoporous molecular sieve through intra-particle diffusion, and then combine with the O^−^ ionized by the silanol.

## 4. Conclusions

In the present work, a recyclable diatomite-based mesoporous materials of MCM-41/co-(PPy-Tp) was successfully synthetised by an effortless and green method and shows excellent adsorption performance for aqueous Hg(ii). The optimal results *via* the RSM and CCD method reveal that the optimal adsorption capacity of MCM-41/co-(PPy-Tp) to Hg(ii) was 537.15 mg g^−1^ at pH = 7.1, *T* = 37.9 °C, *C*_0_ = 45.1 mg L^−1^ and dosage of 0.064 g L^−1^. The adsorption process is fitted welled with the Langmuir and pseudo-second-order models, manifesting that monolayer chemisorption is a rate control step. The adsorption of Hg(ii) onto MCM-41/co-(PPy-Tp) is mainly associated with electrostatic attraction and surface chelation. Besides, the as-developed MCM-41/co-(PPy-Tp) adsorbent displays excellent recyclability and stabilization. The application of MCM-41/co-(PPy-Tp) in the treatment of actual electroplating wastewater exhibits excellent removal ability to Hg(ii) ions. All the results demonstrate that the as-prepared functionalized diatomite-based mesoporous materials of MCM-41/co-(PPy-Tp) is a promising adsorbent for mitigating mercury pollution in water.

## Conflicts of interest

The authors declare that there are no conflicts of interest.

## Supplementary Material

RA-012-D2RA05938J-s001
