# Supplementary material for: Selective adsorption of Hg(ii) with diatomite-based mesoporous materials functionalized by pyrrole–thiophene copolymers: condition optimization, application and mechanism
Source: RSC Adv. 2022 Nov 18;12(51):33160–74. doi: 10.1039/d2ra05938j (PMC9673902; doi:10.1039/d2ra05938j)
Supplement: RA-012-D2RA05938J-s001 [file RA-012-D2RA05938J-s001.pdf]

Electronic Supplementary Material (ESI) for RSC Advances.
This journal is © The Royal Society of Chemistry 2022

Supporting Information

Selective adsorption towards Hg(II) with diatomite-based mesoporous material functionalized by copolymer of pyrrole-thiophene: Condition optimization, application and mechanism

Yu Zhou[a,ξ], Zheng Zeng[a,ξ], Yongfu Guo[a,b,*], Xinyu Zheng[a,*]

[a] Department of Municipal Engineering, Suzhou University of Science and Technology, Suzhou 215009, China

[b] Jiangsu Collaborative Innovation Center of Technology and Material of Water Treatment, Suzhou 215009, Jiangsu, China

[ξ] These authors contributed equally to this work.

[*] Corresponding author.

E-mail address: yongfuguo@163.com (Y. Guo), xinyuzheng@mail.usts.edu.cn (X. Zheng)

1. SEM

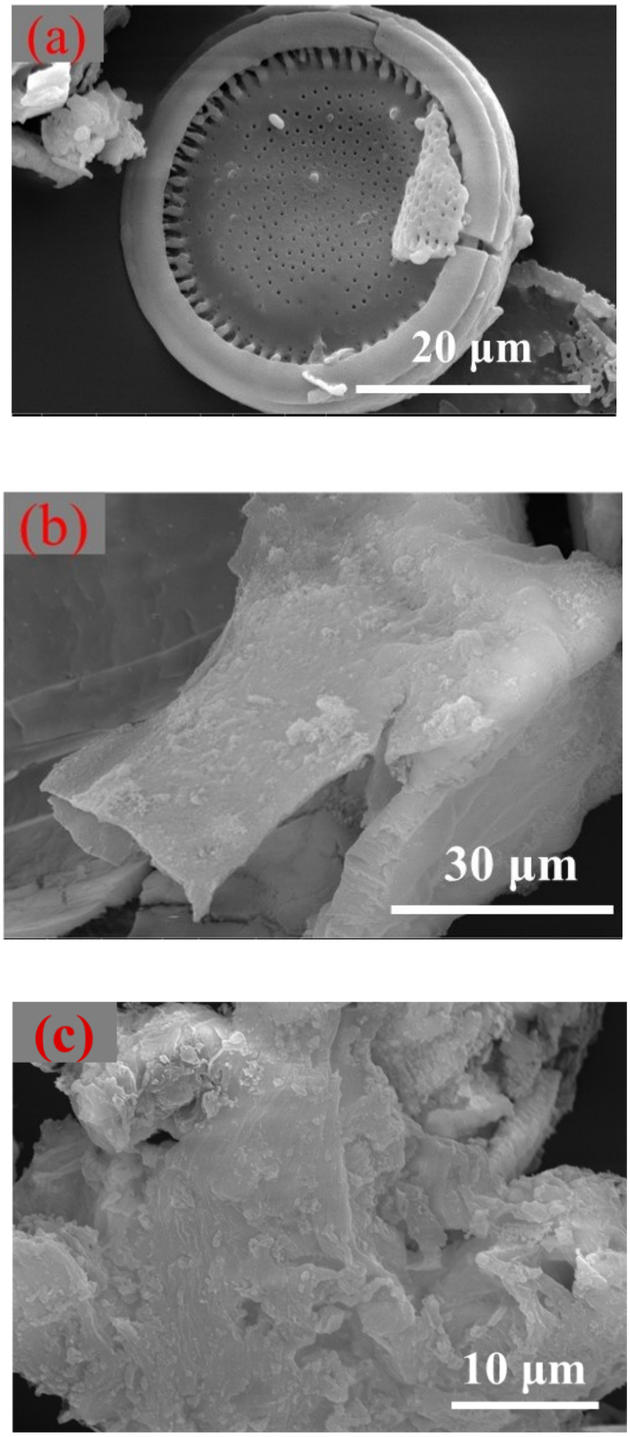

Fig. S1. SEM images of DMTs (a), MCM-41 (b) and MCM-41/co-(PPy-Tp) (c).

2. EDS

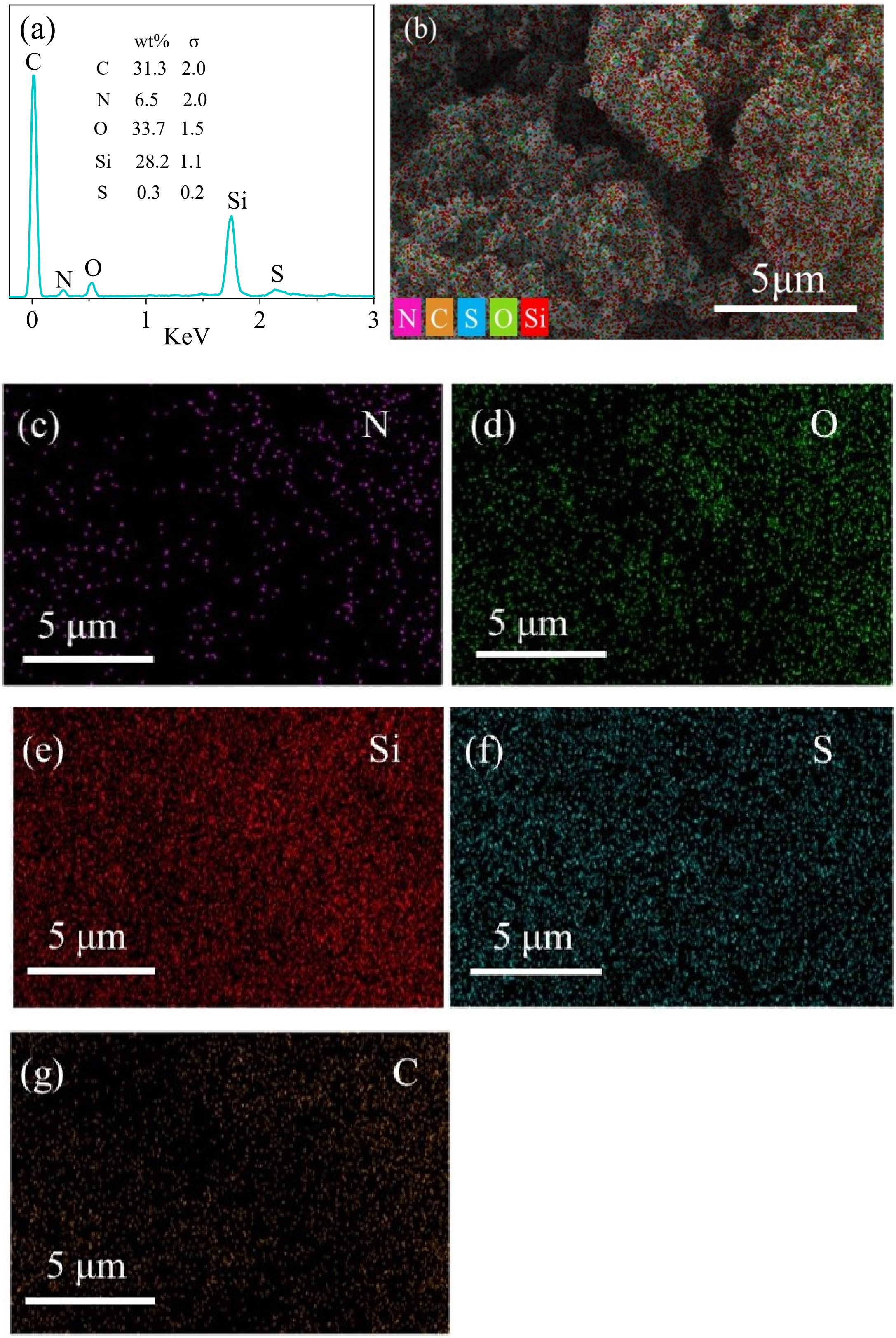

Fig. S2. EDS and mapping of MCM-41/co-(PPy-Tp).

3. Response surface methodology design

Table S1. MCM-41/co-(PPy-Tp) response surface design.

Std.	Run	Variables				q_e
		pH (A)	T (B)	C_0 (C)	Dosage (D)	
1	28	5	25	40	0.06	464
2	24	9	25	40	0.06	499
3	11	5	45	40	0.06	516
4	7	9	45	40	0.06	516
5	23	7	35	20	0.04	439
6	3	7	35	60	0.04	440
7	18	7	35	20	0.08	461
8	22	7	35	60	0.08	492
9	1	5	35	40	0.04	452
10	15	9	35	40	0.04	472
11	16	5	35	40	0.08	466
12	25	9	35	40	0.08	497
13	8	7	25	20	0.06	481
14	27	7	45	20	0.06	492
15	9	7	25	60	0.06	484
16	12	7	45	60	0.06	505
17	4	5	35	20	0.06	478
18	10	9	35	20	0.06	506
19	20	5	35	60	0.06	499
20	19	9	35	60	0.06	510
21	13	7	25	40	0.04	435
22	2	7	45	40	0.04	465
23	29	7	25	40	0.08	453
24	17	7	45	40	0.08	503
25	6	7	35	40	0.06	528
26	5	7	35	40	0.06	536
27	21	7	35	40	0.06	535
28	26	7	35	40	0.06	529
29	14	7	35	40	0.06	532

Table S2. Quadratic model ANOVA analysis.

Source	Sum of squares	df	Mean square	F-value	P-value	
Model	171464.1	14	12247	2184.9	< 0.0001	Significant
A-pH	98176.04	1	98176.04	17514	< 0.0001	
B-T	4401.04	1	4401.04	785.12	< 0.0001	
C-C_0	7455.38	1	7455.375	1330	< 0.0001	
D-Dosage	1335.04	1	1335.042	238.16	< 0.0001	
AB	3751.56	1	3751.56	669.3	< 0.0001	
AC	4522.56	1	4522.56	806.8	< 0.0001	
AD	1785.06	1	1785.06	318.44	< 0.0001	
BC	770.062	1	770.06	137.37	< 0.0001	
BD	1387.56	1	1387.56	247.53	< 0.0001	
CD	18.07	1	18.07	3.22	0.0928	
A^2	37697.86	1	37697.86	6725.1	< 0.0001	
B^2	2714.36	1	2714.36	484.23	< 0.0001	
C^2	11328.57	1	11328.57	2021	< 0.0001	
D^2	9589.36	1	9589.36	1710.7	< 0.0001	
Residual	84.083	15	5.61			
Lack of Fit	74.75	10	7.48	4.004464	0.0395	Insignificant
Pure Error	9.33	5	1.867			
Cor Total	171548.2	29				

4. Models of adsorption kinetics

Pseudo-first-order model:

$$\ln\left(q_e - q_t\right) = lnq_e - k_1 t \tag{S1}$$

Pseudo-second-order model:

$$q_t = K_d t^{1/2} + C \tag{S2}$$

Intra-particle diffusion model:

$$q_t = K_d t^{1/2} + C \tag{S3}$$

Elovich model:

$$q_t = \frac{1}{\beta} ln(\alpha\beta) + \frac{1}{\beta} lnt \tag{S4}$$

Double constant equations models:

$$lnq_t = lnA + Blnt \tag{S5}$$

where, q_e (mg/g) is the equilibrium adsorption capacity; q_t (mg/g) is the adsorption capacity at time t (min); K_1 (min^{-1}) is the first order adsorption rate constant; K_2 (mg/(g·min)) is the second order adsorption rate constant; K_d (mg/(g·$min^{0.5}$)) is the intraparticle diffusion rate constant; α and β are the constants of Elovich, which represent the initial adsorption rate constant and desorption rate constant, respectively (mg/(g·min) and g/mg); α and β are equation parameters and and C (mg/g) is the boundary layer thickness.

5. Adsorption isothermal models

Langmuir model (Eq. (S6)) is usually used to describe single-layer adsorption and indicates that adsorption occurs on the surfaces with the same sites. Freundlich model (Eq. (S7)) is used to describe the adsorption process on non-uniform surfaces and generally

considers the possibility of single-layer adsorption and multi-layer adsorption. Temkin isotherm model (Eq. (S8)) and Dubinin-Radushkevich (D-R) model (Eq. (S9)) were also used to fit mercury adsorption process.

$$\frac{C_e}{q_e} = \frac{C_e}{Q_m} + \frac{1}{Q_m K_L} \tag{S6}$$

$$\ln q_e = \ln K_f + \frac{1}{n} \ln C_e \tag{S7}$$

$$q_e = B \ln K_T + B \ln C_e \tag{S8}$$

$$\ln q_e = \ln Q_m - \beta \varepsilon^2 \tag{S9}$$

$$E = \frac{1}{(2\beta)^{1/2}} \tag{S10}$$

where, K_L is adsorption constant. Q_m is maximum adsorption capacity (mg/g). K_f is Freundlich constant. n is adsorption intensity. B and K_T are Temkin isotherm constants. ε is Polanyi potential and β is D-R isotherm constant. E (kJ/mol) is the average adsorption energy per mole of metal ions transferring from liquid phase to the surface of the adsorbent.

6. Model of Thermodynamics

$$\Delta G^0 = -RT \ln K_R \tag{S10}$$

$$\ln K_R = \frac{\Delta S^0}{R} - \frac{\Delta H^0}{RT} \tag{S11}$$

where, K_R is equilibrium constant; R is gas constant (8.314 J/mol/K).
